# Rank Signaling Links the Development of Invariant γδ T Cell Progenitors and Aire^+^ Medullary Epithelium

**DOI:** 10.1016/j.immuni.2012.01.016

**Published:** 2012-03-23

**Authors:** Natalie A. Roberts, Andrea J. White, William E. Jenkinson, Gleb Turchinovich, Kyoko Nakamura, David R. Withers, Fiona M. McConnell, Guillaume E. Desanti, Cecile Benezech, Sonia M. Parnell, Adam F. Cunningham, Magdalena Paolino, Josef M. Penninger, Anna Katharina Simon, Takeshi Nitta, Izumi Ohigashi, Yousuke Takahama, Jorge H. Caamano, Adrian C. Hayday, Peter J.L. Lane, Eric J. Jenkinson, Graham Anderson

**Affiliations:** 1 MRC Centre for Immune Regulation, University of Birmingham, Birmingham, B15 2TT, UK; 2 London Research Institute, Cancer Research UK, London, WC2A 3LY, UK; 3 Peter Gorer Department of Immunobiology, Kings College at Guy's Hospital, London, SE1 9RT, UK; 4 IMBA, Institute of Biotechnology of the Austrian Academy of Sciences, 1030 Vienna, Austria; 5 Human Immunology Unit, Nuffield Department of Medicine, University of Oxford, Oxford, OX3 9DS, UK; 6 Division of Experimental Immunology, Institute for Genome Research, University of Tokushima, Tokushima 770-8503, Japan

## Abstract

The thymic medulla provides a specialized microenvironment for the negative selection of T cells, with the presence of autoimmune regulator (Aire)-expressing medullary thymic epithelial cells (mTECs) during the embryonic-neonatal period being both necessary and sufficient to establish long-lasting tolerance. Here we showed that emergence of the first cohorts of Aire^+^ mTECs at this key developmental stage, prior to αβ T cell repertoire selection, was jointly directed by Rankl^+^ lymphoid tissue inducer cells and invariant Vγ5^+^ dendritic epidermal T cell (DETC) progenitors that are the first thymocytes to express the products of gene rearrangement. In turn, generation of Aire^+^ mTECs then fostered Skint-1-dependent, but Aire-independent, DETC progenitor maturation and the emergence of an invariant DETC repertoire. Hence, our data attributed a functional importance to the temporal development of Vγ5^+^ γδ T cells during thymus medulla formation for αβ T cell tolerance induction and demonstrated a Rank-mediated reciprocal link between DETC and Aire^+^ mTEC maturation.

## Graphical abstract

**Graphical Abstract:**

**Figure:**
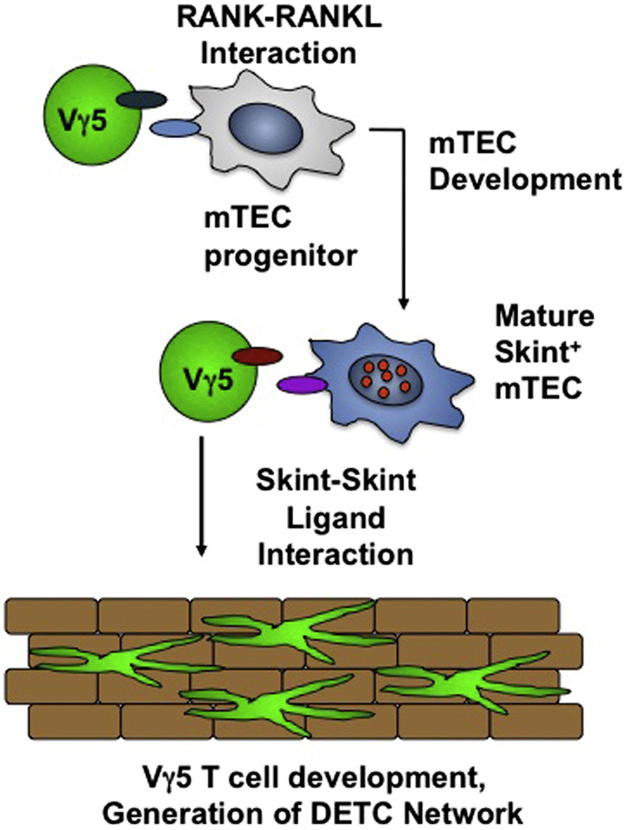

**Highlights:**

► Invariant Vγ5^+^ thymocytes regulate formation of Aire^+^ medullary thymic epithelium ► Generation of an invariant Vγ5^+^ T cell population requires thymus medulla development ► Skint-1-mediated Vγ5^+^ thymocyte development is Aire independent ► Dependency on *Tnfrsf11a* links γδ T cell and medullary epithelium development

## Introduction

Shaping of the immature αβTCR repertoire within the thymus is necessary to generate a naive T cell pool biased toward the recognition of self MHC molecules (positive selection) but purged (by negative selection) of potentially autoreactive specificities (Boehm, 2011). These αβ T cell selection events appear to be anatomically compartmentalized in the thymus (Takahama, 2006), in keeping with the finding that intrathymic microenvironments contain distinct, functionally specialized epithelial cell types that regulate thymic selection (Jiang et al., 1995; Surh et al., 1992). Although the epithelial cells in the thymic cortex play a key role in the positive selection and continued maturation of CD4^+^CD8^+^ thymocytes able to interact with self-peptide-MHC complexes (Gommeaux et al., 2009; Honey et al., 2002; Murata et al., 2007; Nitta et al., 2010; Ripen et al., 2011), epithelial cells and dendritic cells (DCs) in the thymic medulla play a key role in negative selection, by which thymocytes bearing strongly self-reactive αβTCRs are eliminated from the developing αβ T cell repertoire (Kyewski and Klein, 2006). In particular, medullary thymic epithelial cells (mTECs), including those expressing the *Aire* gene (Björses et al., 1998; Heino et al., 1999, 2000), influence negative selection in several ways (Anderson et al., 2002; Derbinski et al., 2005; Liston et al., 2003), including expression of a wide array of tissue-restricted antigens for direct and indirect antigen presentation to newly selected thymocytes (Gallegos and Bevan, 2004), and the regulation of intrathymic DC positioning via Aire-dependent XCL1 expression (Lei et al., 2011).

Normal mTEC development depends on NF-κB signaling, as shown by medullary abnormalities and tolerance breakdown in mice deficient in RelB (Burkly et al., 1995; Naspetti et al., 1997), Traf6 (Akiyama et al., 2005), and Nik (Kajiura et al., 2004). Moreover, mTEC maturation requires hematopoietic cell cross-talk (Shores et al., 1991), which involves signaling through various mTEC-expressed TNF receptor superfamily (TNFRSF) members (Boehm et al., 2003; Zhu and Fu, 2008). Regarding the Aire^+^ mTEC subset, which first emerges around embryonic day (E) 16 of gestation (Gäbler et al., 2007; White et al., 2008; Zuklys et al., 2000), Rank (TNFRSF11a, CD265, TRANCER) plays a key role (Rossi et al., 2007), whereas in the steady-state adult thymus, synergy between Rank and CD40 regulates Aire^+^ mTEC development (Akiyama et al., 2008; Hikosaka et al., 2008; Irla et al., 2008). Importantly, by controlling and limiting the temporal deletion of Aire^+^ mTECs to either neonatal or adult thymus, a recent study showed that Aire^+^ mTECs in the embryonic and neonatal period are both essential and sufficient to establish long-term T cell tolerance (Guerau-de-Arellano et al., 2009). Thus, the development of the first cohorts of Aire^+^ mTECs from Rank-expressing mTEC progenitors is a key step in the avoidance of autoimmunity. Whereas Rank ligand (Rankl)-expressing, positively selected thymocytes play a role in the development of Aire^+^ mTECs in the adult thymus (Hikosaka et al., 2008), we showed that Rankl^+^ lymphoid tissue inducer (LTi) cells, master regulators of lymphoid tissue organogenesis (Eberl et al., 2004; Finke et al., 2002; Mebius et al., 1997; Sun et al., 2000), are a key determinant of Rank-dependent thymus medulla development in the embryo (Rossi et al., 2007). Taken together with the key role of the first Aire^+^ mTEC cohorts in tolerance induction (Guerau-de-Arellano et al., 2009), these findings support a preemptive role for innate LTi cells, in which Aire^+^ mTECs develop independently of and prior to αβ T cell-positive selection, ensuring that they are in place to induce tolerance in the nascent αβ T cell repertoire. However, although Rankl^+^ LTi induce mTEC differentiation, the presence of Aire^+^ mTECs in the developing embryonic thymus of LTi-deficient *Rorc*^−/−^ mice at a stage prior to αβ T cell selection (White et al., 2008) suggests that additional embryonic cell types play a distinct role in establishing the medullary microenvironments that ensure T cell tolerance induction.

Prior to αβ T cells, the embryonic thymus generates T cells that express the γδTCR (Havran and Allison, 1988, 1990; Pennington et al., 2003). Indeed, the first appearance of Aire^+^ mTECs (White et al., 2008; Zuklys et al., 2000) coincides with that of progenitors of Vγ5^+^ dendritic epidermal T cells (DETCs), a subset of invariant intraepithelial lymphocytes (IELs) whose development is uniquely linked to the embryonic thymus (Asarnow et al., 1988; Ikuta et al., 1990; Mallick-Wood et al., 1998). Although the invariant nature of the Vγ5Vδ1TCR repertoire expressed by DETC progenitors distinguishes them from diverse αβ T cell precursors, several studies demonstrate a role for selection events during intrathymic DETC development (Passoni et al., 1997; Xiong et al., 2004). In particular, Skint-1, an immunoglobulin superfamily member expressed by TECs, is essential for the selection and generation of the monoclonal DETC compartment (Barbee et al., 2011; Boyden et al., 2008; Lewis et al., 2006). Furthermore, DETC development is followed within a few days by the emergence of γδ T cells with diverse TCRs that, like αβTCRs, may need selective focusing to avoid autoimmune pathology. Thus, intrathymic epithelial microenvironments may play distinct and critical roles in the generation and selection of both diverse and invariant γδ T cell subsets.

Given the importance of tolerance induction in the neonatal period, we sought to examine possible links between the initial formation of intrathymic microenvironments that impose tolerance during this early developmental window in the immune system and the early appearance of γδ T cells. We show that the scheduled early development of invariant Vγ5^+^ DETC progenitors makes a distinct contribution to the development of Aire^+^ mTEC maturation. Moreover, we show a link between Rank-mediated Aire^+^ mTEC development and the functional maturation of Vγ5^+^ DETC progenitors via their expression of *Skint1*. This link between Vγ5^+^ DETC thymocyte progenitor maturation and Rank-mediated mTEC development was further underlined by a block in the intrathymic development of Vγ5^+^ progenitors in the thymus of Rank-deficient (*Tnfrsf11a*^−/−^) mice at the γδTCR^lo^CD45RB^lo^ stage, which further manifested as diminished numbers of mature epidermal-resident DETCs and the loss of an invariant Vγ5^+^ DETC repertoire, in *Tnfrsf11a*^−/−^ neonatal epidermis. However, despite these links between Skint-1 and Aire^+^ mTECs, *Skint1* expression was found to be Aire independent, and intrathymic Vγ5^+^ thymocyte development and the emergence of invariant Vγ5^+^ DETCs proceeded normally in *Aire*^−/−^ mice. Collectively, our data have identified the key cellular components that regulate the emergence of Aire^+^ mTECs at initial stages of thymus development and defined a critical role for Rank-Rankl-mediated interactions between fetal γδ T cell progenitors and mTECs that reciprocally regulate their maturation.

## Results

### Vγ5^+^ DETC Progenitors Associate with Fetal mTECs

**Figure 1:**
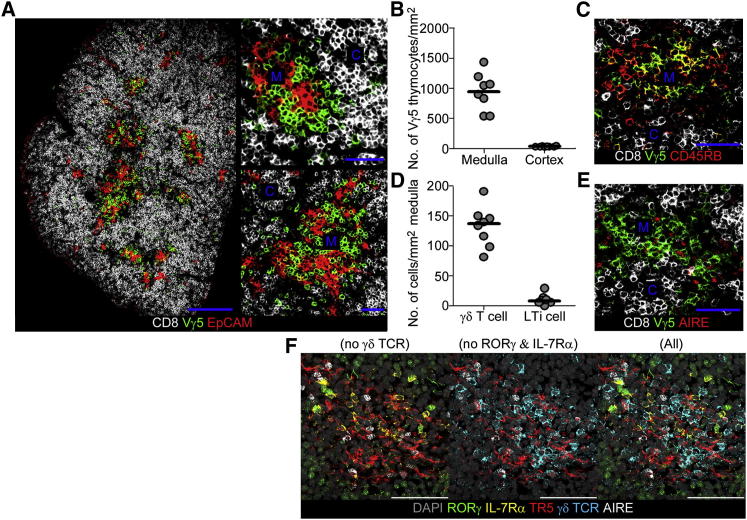
In Fetal Thymus, Vγ5^+^ Thymocytes Associate with Developing Aire-Expressing Medullary Environments (A) E17 thymus lobes from B6 mouse embryos were stained with antibodies to CD8 (white), Vγ5TCR (green), and EpCAM1 (red). CD8 expression denotes the cortical areas containing CD4^+^CD8^+^ thymocytes. (B) Quantitation of the distribution of Vγ5^+^ thymocytes in cortical and medullary areas of E17 thymic sections. Each point represents an individual thymus lobe, and horizontal lines on the graph represent the mean. (C) An image of a frozen section of E17 thymus stained for CD8 (white), CD45RB (red), and Vγ5TCR (green). (D) Quantitation of the frequency of RORγ^+^IL-7Rα^+^ LTi and Vγ5TCR^+^ thymocytes in E17 thymic medullary regions, with each point representing an individual thymus lobe, and horizontal lines on the graph representing the mean. (E) Image of an E17 frozen thymus section for CD8 (white), Aire (red), and Vγ5TCR (green). (F) Confocal image of a medullary area within an E17 thymus section stained with antibodies to RORγ (green), IL-7Rα (yellow), TR5 (red), γδTCR (blue), and Aire (white). Nuclei are stained with DAPI (gray). For clarity, a series of images with various marker combinations is shown.

We previously showed that generation of the first cohorts of Aire^+^ mTECs in the embryonic thymus involves signals from Rankl^+^ LTi cells (Rossi et al., 2007), a population originally identified as a key player in the development of stromal microenvironments within secondary lymphoid tissues such as lymph node (Mebius, 2003). However, when we analyzed thymus medulla formation in *Rorc^−/−^* embryonic mice lacking LTi, we found Aire^+^ mTECs still present, albeit at reduced numbers (White et al., 2008). This nonessential role for LTi cannot be explained by Rankl provision by positively selected αβTCR^+^ thymocytes, because Aire^+^ mTECs are present in *Rorc*^−/−^ embryonic mice prior to αβ T cell selection (White et al., 2008). To investigate the possibility that previously unidentified cell types influence initial thymus medulla formation in the developing embryonic thymus, we screened the cellular makeup of medullary areas in E17 thymus tissue sections. Of the cell types analyzed, and consistent with an earlier report (Farr et al., 1990), a pan-γδTCR antibody defined a striking concentration of γδTCR^+^ thymocytes within developing embryonic EpCAM1^+^ medullary areas (not shown). Moreover, by using a Vγ5TCR-specific antibody, we found that essentially all of the medullary-resident γδTCR^+^ thymocytes at this stage expressed the Vγ5TCR (Figures 1A and 1B), representing DETC progenitors. Further analysis showed that some medullary-resident Vγ5^+^ T cells expressed high levels of CD45RB (Figure 1C), a maturational marker of intrathymic DETC progenitors (Lewis et al., 2006), suggestive of a link between thymus medulla development and DETC progenitor maturation. Confocal analysis of embryonic thymus sections stained to reveal the localization and frequency of Vγ5^+^ thymocytes and RORγ^+^CD4^+^CD3^−^IL-7Rα^+^ LTi showed an abundance of Vγ5^+^ thymocytes relative to LTi cells, with quantitative analysis indicating an approximate 100:1 ratio for Vγ5^+^ thymocytes:LTi within medullary areas (Figure 1D). Importantly, dual staining with antibodies to Aire and Vγ5TCR revealed individual medullary areas containing both Aire^+^ mTECs and Vγ5^+^ thymocytes (Figure 1E), whereas staining with Aire and pan-γδTCR antibodies together with IL-7Rα and RORγ antibodies to reveal IL-7Rα^+^RORγ^+^ LTi demonstrated the presence of both γδTCR^+^ thymocytes and LTi within individual Aire-expressing medullary areas (Figure 1F). Analysis of the anatomical distribution of LTi and Vγ5 thymocytes in relation to Aire^+^ mTECs in thymus tissue sections failed to reveal a defined pattern to the distribution of these cells within multiple medullary areas (not shown). However, although there may be no difference in the topological positioning of these cells in the thymic medulla, the combined presence of Vγ5^+^ thymocytes and LTi cells within individual medullary areas suggests that they act collectively to influence mTEC development. Taken together, these results indicate that in the fetal thymus, in addition to the presence of LTi cells, there is an anatomical association between fetal-specific Vγ5 thymocytes and thymic medullary epithelial cells.

### Vγ5^+^ Thymocytes Express Rankl and Drive Aire^+^ mTEC Development

**Figure 2:**
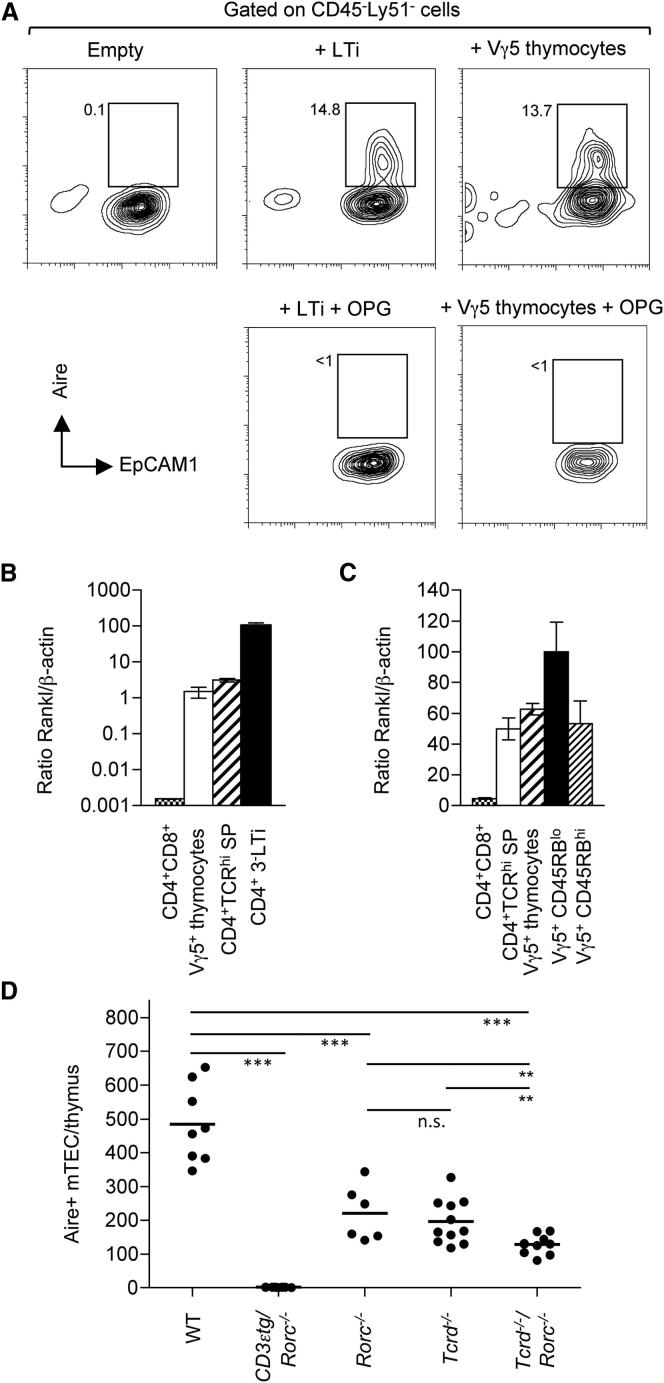
Vγ5^+^ Thymocytes Express Rankl and Induce Aire^+^ mTEC Development (A) Reaggregate thymus organ cultures were prepared from either 2 dGuo-treated thymic stromal cells alone (left), or with added LTi (middle) or added Vγ5^+^ fetal thymocytes (right). In some cultures, recombinant OPG was added at a final concentration of 10 μg/ml (bottom). After 5 days, cultures were disaggregated, and FACS analysis is shown for EpCAM1 and nuclear Aire, gated on CD45^−^Ly51^−^ cells. Numbers indicate percentages of cells. (B and C) Quantitative PCR analysis of *Rankl* is shown for thymocyte populations and total Vγ5^+^ thymocytes (B) and *Rankl* expression in thymocytes and CD45RB subsets of Vγ5^+^ thymocytes (C). Levels of mRNA were normalized to *ACTB* (β-actin). (D) Cell numbers of Aire^+^EpCAM1^+^Ly51^−^ mTECs within freshly disaggregated E17 thymus lobes of the indicated mouse strains. Each point represents a single thymus lobe, with horizontal lines representing the mean. Asterisks indicate statistically significant differences; ^∗∗∗^p < 0.001, ^∗∗^p < 0.006, n.s., not significant.

To investigate the possibility that Vγ5^+^ DETC thymocyte progenitors influence the formation of embryonic mTEC microenvironments, we first made reaggregate thymus organ cultures (RTOCs) by using 2 dGuo fetal thymus lobes, known to contain the Rank^+^ progenitors of Aire^+^ mTECs (Rossi et al., 2007), into which either purified Vγ5^+^ thymocytes or LTi were added. After 5 days, RTOCs were disaggregated and analyzed by flow cytometry for the appearance of mature EpCAM1^+^Ly51^−^Aire^+^ mTECs. Consistent with our previous observations that mTEC progenitor development depends upon hematopoietic cell crosstalk (Rossi et al., 2007), Aire^+^ mTECs were absent in RTOCs initiated without added hematopoietic cells (Figure 2A, left) but were found to be present after the addition of LTi (Figure 2A, middle). Strikingly, analysis of RTOCs initiated with Vγ5^+^ thymocytes (Figure 2A, right) also induced the emergence of a defined cohort of EpCAM1^+^Ly51^−^Aire^+^ mature mTECs, providing direct evidence that DETC progenitors can influence the formation of embryonic medullary thymic microenvironments. Despite an approximate 100-fold difference in Rankl expression in LTi cells and Vγ5^+^ thymocytes (Figure 2B), both cell types induced a similar proportion of Aire^+^ mTECs in RTOC experiments (Figure 2A, middle and right). Importantly, RTOC experiments in which Rank-Rankl interactions were inhibited by addition of the soluble decoy receptor OPG completely abrogated Aire^+^ mTEC development induced by both Vγ5^+^ thymocytes and LTi cells (Figure 2A). Collectively, these experiments demonstrate the potency of Rank signaling in mTEC development and directly show that Rankl expression by Vγ5^+^ thymocytes and LTi cells underpins the ability of these cells to induce Aire^+^ mTEC development.

Given that previous experiments highlighted the particular importance of Rank-Rankl signaling in embryonic Aire^+^ mTEC maturation, we next analyzed *Rankl* mRNA expression in a variety of thymic populations by qPCR. In contrast to CD4^+^CD8^+^ thymocytes that are known to lack *Rankl* expression (Hikosaka et al., 2008), purified Vγ5^+^ thymocytes and LTi cells were both found to expressed readily detectable levels of *Rankl* (Figure 2B). Although *Rankl* expression by Vγ5^+^ thymocytes was found to be lower than that of LTi, it was comparable to that of positively selected CD4^+^αβTCR^hi^ thymocytes (Figure 2B), which is sufficient to drive Aire^+^ mTEC development in the adult (Hikosaka et al., 2008). Thus, *Rankl* expression by Vγ5TCR^+^ thymocytes correlates well with our finding that they can induce Rank-dependent Aire^+^ mTEC maturation. Interestingly, qPCR analysis of separated CD45RB^lo^ and CD45RB^hi^ subsets of Vγ5^+^ thymocytes showed that both expressed *Rankl*, with a slightly higher levels of expression being detected in CD45RB^lo^ cells (Figure 2C). This finding is of significance because it suggests that unlike αβT cells, which rely on positive selection to reach the Rankl^+^ stage and influence mTECs (Hikosaka et al., 2008), Vγ5 T cells are equipped with Rankl at an immature stage, indicating that Skint-1-mediated differentiation does not determine the ability of γδ T cells to influence the thymic medulla. Combined with functional data on the importance of Rankl in Aire^+^ mTEC development, this suggests that both immature and mature Vγ5^+^ thymocytes have the potential to influence mTEC development. Collectively, these findings demonstrate that intrathymic Vγ5^+^ DETC progenitors express *Rankl*, are accumulated in developing medullary areas of the fetal thymus, and can induce the maturation of mTEC progenitors into mature Aire^+^ mTECs.

We next analyzed the frequency of Aire^+^ mTECs within the fetal thymus at E17 of gestation in WT mice and in mice individually deficient in either LTi (*Rorc*^−/−^) or γδ T cells (*Tcrd*^−/−^) or both (*Rorc^−/−^* × *Tcrd^−/−^*). As expected, mice lacking the capacity to provide hematopoietic crosstalk signals via combined blockade of T cell development/LTi development (CD3εtg26/*Rorc*^−/−^ mice) displayed a complete absence of Aire^+^ mTECs (Figure 2D). By contrast, both *Tcrd*^−/−^ and *Rorc*^−/−^ single mutant mice showed a partial, but statistically significant, defect in the generation of Aire^+^ mTECs as compared to WT controls (Figure 2D). Interestingly, analysis of *Rorc^−/−^* × *Tcrd^−/−^* double-deficient mice revealed a further reduction in the frequency of Aire^+^ mTECs compared to *Tcrd*^−/−^ and *Rorc*^−/−^ single mutants. Thus, these findings indicate that although LTi and γδ T cells are required in order to generate Aire^+^ mTECs at a normal frequency, the presence of small numbers of Aire^+^ mTECs in their combined absence, which contrasts to the total absence of Aire^+^ mTECs in CD3εtg26/*Rorc*^−/−^ mice, suggests that additional hematopoietic cell types can also involved.

### Skint-1 Is Expressed by Mature mTECs and Is Induced by Rank Signaling

**Figure 3:**
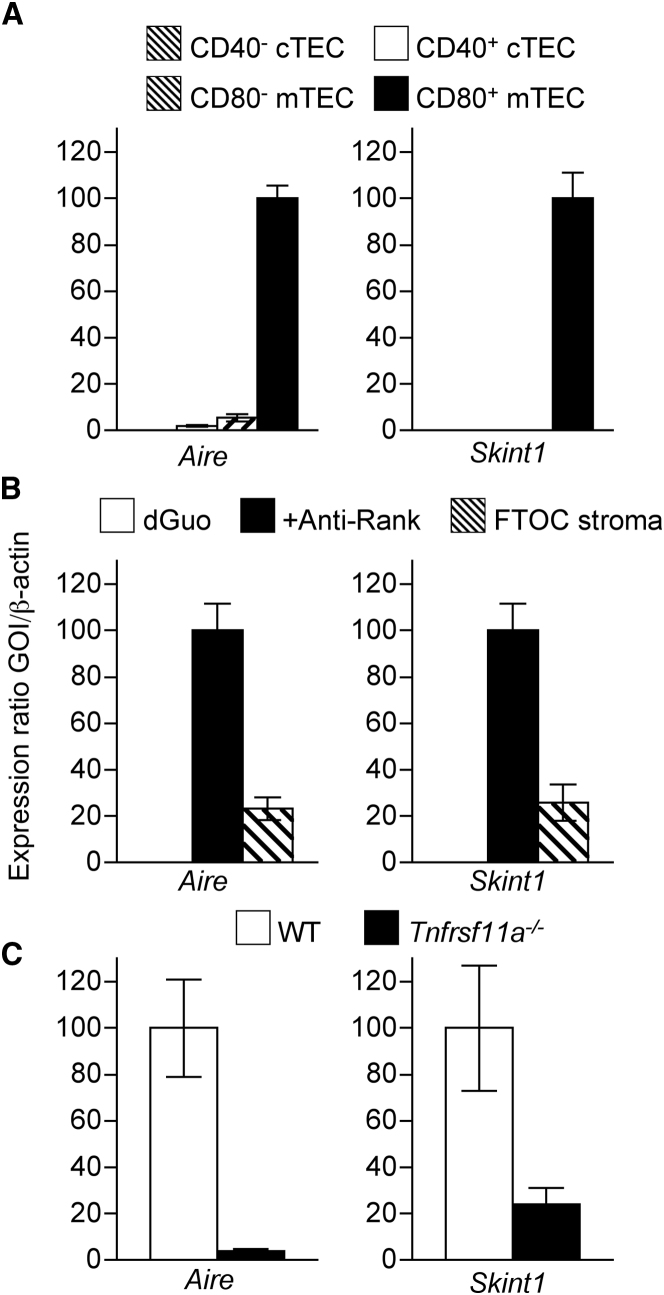
*Skint1* Expression Maps to CD80^+^ mTECs and Is Regulated by Rank Signaling (A) Quantitative PCR analysis of *Aire* and *Skint1* in freshly isolated TEC subsets. (B) 2-dGuo-treated FTOC cultured in the presence (black bars) or absence (white bars) of Rank antibody were analyzed by qPCR for expression of *Aire* and *Skint1*. Levels of expression in total CD45^−^ FTOC stroma (hatched bars) are shown for comparison. (C) qPCR analysis of the indicated genes in CD45^−^ cells isolated from WT (white bars) and *Tnfrsf11a*^−/−^ (black bars) E15 thymus lobes, established in FTOC for 7 days. In all cases, levels of mRNA were normalized to β-actin.

Intrathymic development of invariant Vγ5^+^ thymocyte progenitors and the generation of an invariant Vγ5^+^ DETC population in the epidermis depends upon thymic stromal cell expression of Skint-1, an Ig superfamily member expressed by mTECs (Lewis et al., 2006). To investigate the possible link between this thymic stromal cell expression of Skint-1, the medullary accumulation of Vγ5^+^ thymocytes, and Aire^+^ mTEC development, we further analyzed *Skint1* expression in embryonic cTEC and mTEC subsets (Shakib et al., 2009), including immature CD80^−^ and mature CD80^+^ mTEC populations shown previously to have a direct precursor-product relationship, with the latter containing Aire^+^ cells (Gäbler et al., 2007; Gray et al., 2007; Rossi et al., 2007). As expected, *Aire* expression was limited to mature CD80^+^ mTECs (Figure 3A), but of note this restricted pattern mirrored that of *Skint1*, which was undetectable in immature and mature stages of the cTEC lineage and in immature CD80^−^ mTECs (Figure 3A).

Given our report of a key role for Rank in the development of fetal mTECs, we analyzed its importance in the regulation of *Skint1.* When we stimulated the development of Aire^+^ mTECs in dGuo-treated FTOCs with agonistic Rank antibodies, the induction of *Aire* expression (Figure 3B) was accompanied by the induction of *Skint1* expression, with unstimulated dGuo-treated FTOCs showing an absence of *Skint1* expression (Figure 3B). In addition, in comparison to levels seen in WT littermate controls, analysis of CD45^−^ thymic stromal cell populations from fetal *Tnfrsf11a*^−/−^ mice showed a dramatic reduction in expression of both *Aire* and *Skint1* (Figure 3C). Thus, our findings that *Skint1* expression in the mTEC lineage is restricted to more mature CD80^+^ mTECs and is linked to Rank-mediated signaling in mTECs further highlight a potential reciprocal link in the development of Aire^+^ mTECs and Vγ5^+^ T cells.

### Rank Regulates Vγ5^+^ Thymocyte Development and the Emergence of an Invariant DETC Repertoire

**Figure 4:**
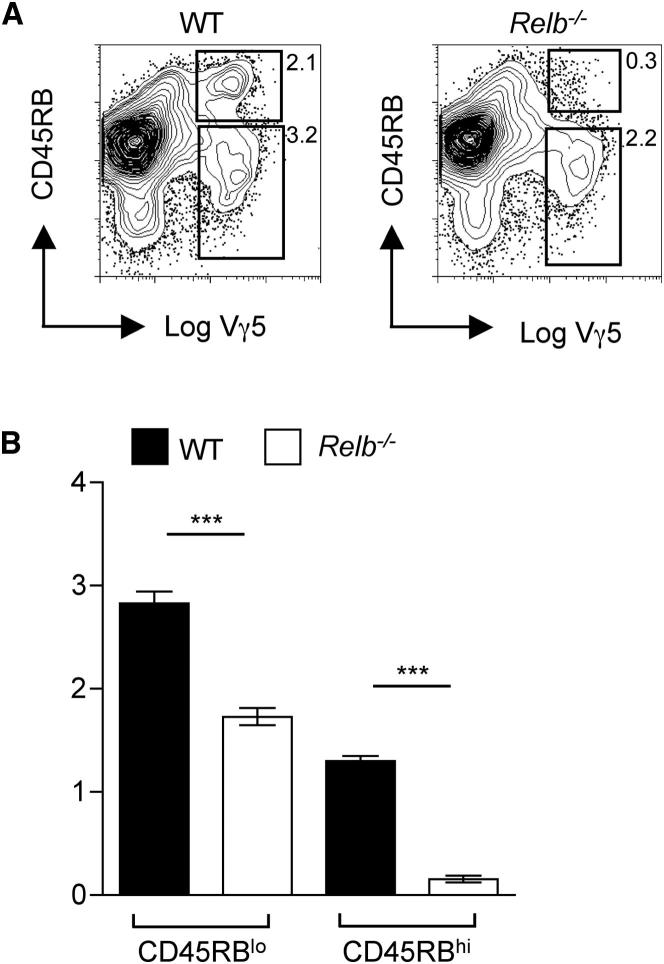
Defective Intrathymic Maturation of Vγ5^+^ DETC Progenitors in *Relb*-Deficient Mice (A) Analysis of Vγ5TCR^+^ thymocyte maturation in WT and *Relb*-deficient E15 thymus lobes placed in FTOCs for 5 days. Numbers indicate the percentages of cell populations. (B) Percentages of immature Vγ5^+^CD45RB^lo^ and mature Vγ5^+^CD45RB^hi^ thymocytes in WT (black bars) and *Relb*-deficient (white bars) FTOCs. A minimum of six mice were analyzed, and an unpaired Student's two-tailed t test was performed with asterisks signifying a significant difference, where p < 0.0001.

To study the potential link between Aire^+^ mTECs and Vγ5^+^ DETC progenitors, we initially studied the embryonic thymus of *Relb*^−/−^ mice, a transcription factor in the alternative NF-κB signaling pathway that is downstream of several TNFSF-R family members including CD40 and Rank, known regulators of mTEC maturation. Consistent with this, and as previously reported, Aire^+^ mTECs were absent from the thymus of E17 *Relb*^−/−^ embryos (data not shown). When we analyzed Vγ5^+^ thymocyte development, with upregulation of CD45RB as a marker of maturation (Lewis et al., 2006), we found a statistically significant decrease in both CD45RB^lo^ and CD45RB^hi^ subsets in *Relb*^−/−^ embryonic thymuses compared to WT controls (Figures 4A and 4B). Despite a reduction in both CD45RB subsets, a dramatic skewing in the ratio (approximately 6:1) of WT:*Relb^−/−^* CD45RB^hi^ cells was observed, compared to a ratio of 1.6:1 for WT:*Relb* CD45RB^lo^ cells, suggesting that although RelB may play a subtle role in the initial emergence of Vγ5CD45RB^lo^ cells, it appears critically important in the maturation to the Vγ5^+^CD45RB^hi^ stage.

**Figure 5:**
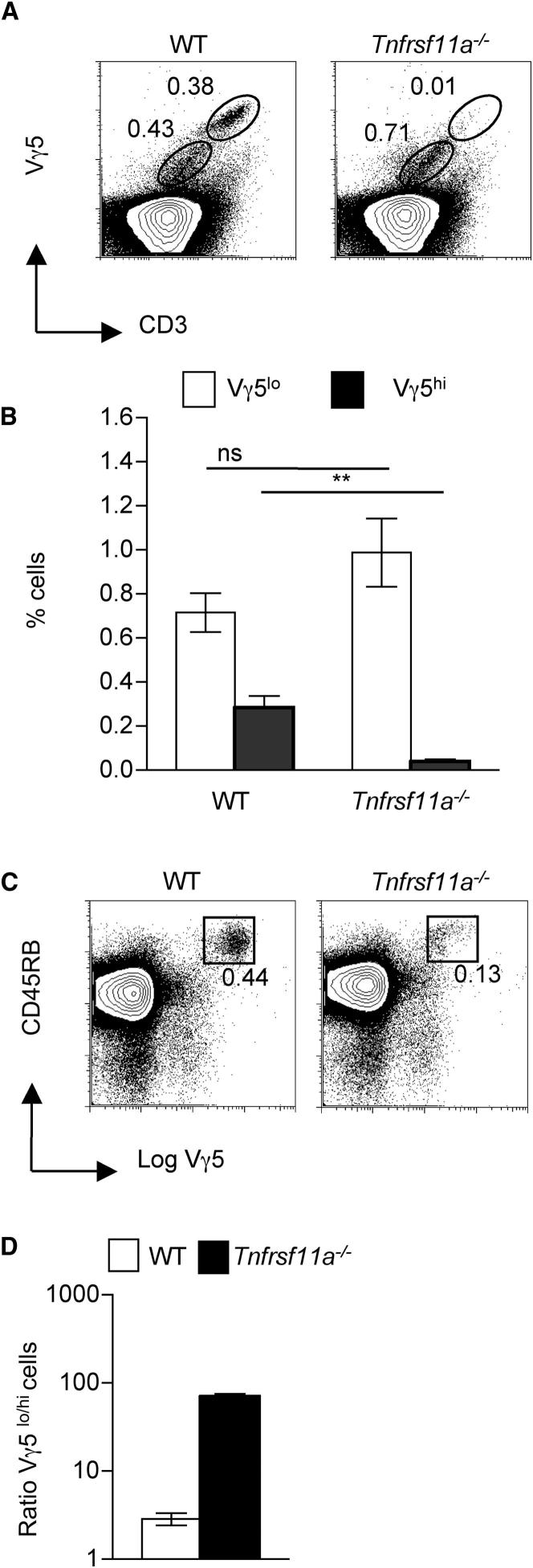
Rank Regulates Intrathymic Maturation of Vγ5^+^ DETC Progenitors (A) Neonatal thymocyte suspensions from WT and *Tnfrsf11a^−/−^* mice were stained with antibodies to the Vγ5TCR and CD3. Circles indicate immature CD3^lo^Vγ5^lo^ and mature CD3^hi^Vγ5^hi^ subsets. (B) Proportions of Vγ5^lo^ (white bars) and Vγ5^hi^ (black bars) thymocytes in WT and *Tnfrsf11a*^−/−^ newborn mice is shown. Averages and standard errors of populations are shown, and asterisks indicate a statistically significant difference where p < 0.01. n.s. is not significant. (C) FACS analysis of thymocytes from neonatal WT and *Tnfrsf11a*^−/−^ mice for expression of CD45RB and Vγ5. Boxes indicate the percentages of Vγ5^hi^CD45RB^hi^ cells. (D) Ratio of Vγ5^lo^Vγ5^hi^ cells in WT (white bars) and *Tnfrsf11a*^−/−^ (black bars) neonatal thymus.

**Figure 6:**
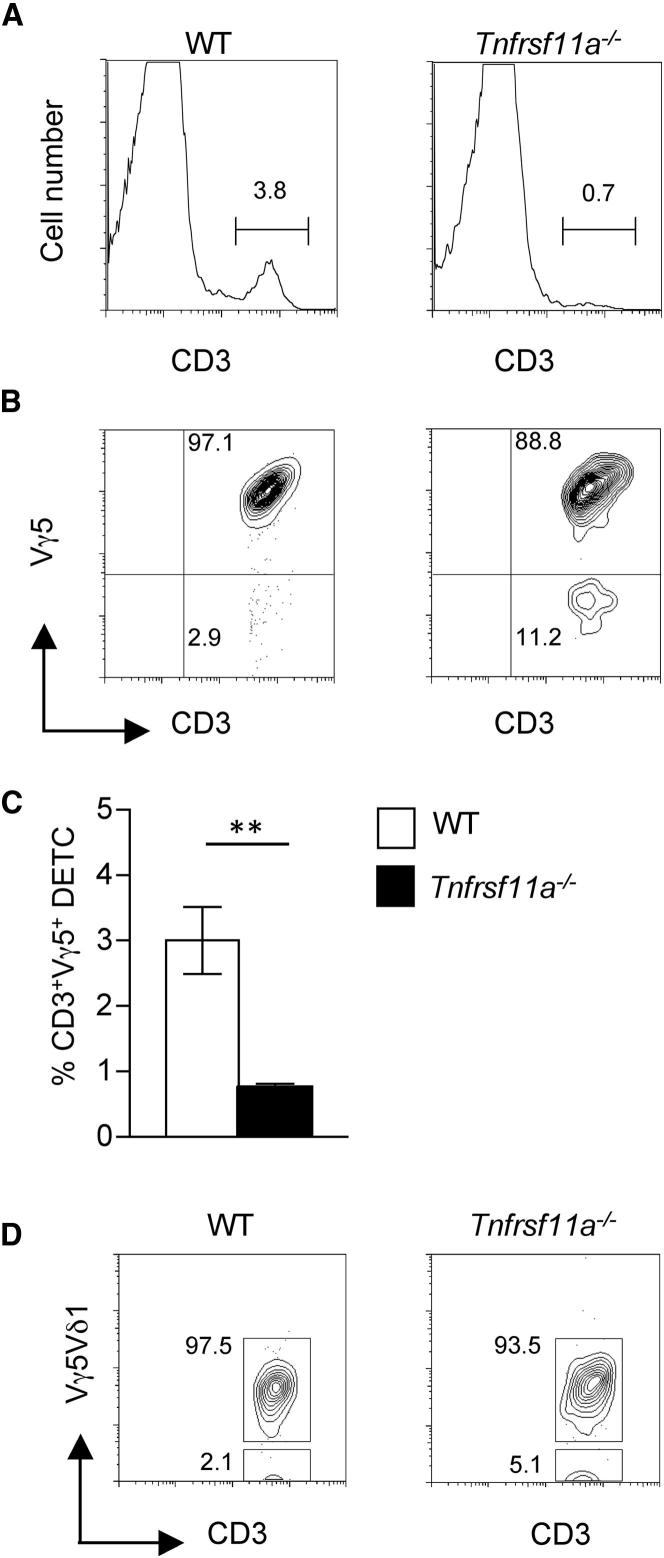
Loss of Rank Impacts Invariant Vγ5^+^ Dendritic Epidermal T Cells (A and B) Epidermal sheets from WT and *Tnfrsf11a*^−/−^ neonatal mice were analyzed by flow cytometry for CD3^+^ T cells (A), together with expression of the Vγ5TCR (B). (C) Frequency of CD3^+^Vγ5^+^ DETCs in WT (white bars) and *Tnfrsf11a*^−/−^ (black bars) neonatal epidermis. Asterisks indicate a statistically significant difference where p < 0.01. (D) Flow cytometric analysis of epidermal sheets from neonatal WT and Rank-deficient mice for expression of CD3 together with the Vγ5Vδ1TCR. Cells are gated on CD3^+^ events, and numbers are percent of CD3^+^ cells.

Given that RelB is downstream of multiple, TNFSF-R family members, we next analyzed by flow cytometry thymocyte suspensions obtained from neonatal *Tnfrsf11a*^−/−^ mice. Again, we found a dramatic reduction in the frequency of cells expressing high levels of both the Vγ5TCR and CD45RB (Figures 5A–5C), which resulted in a skewing in the mature:immature DETC ratio in *Tnfrsf11a*^−/−^ mice (1:100) as compared to WT littermate controls (1:3) (Figure 5D). In addition, analysis of neonatal epidermal preparations showed that *Tnfrsf11a*^−/−^ mice have a dramatic reduction in the frequency of total epidermal CD3^+^ T cells (Figure 6A), as well as a disproportionate reduction in those expressing the Vγ5TCR (Figures 6B and 6C). Further flow cytometric analysis with an antibody that detects the Vγ5Vδ1 γδTCR (clone 17D1) showed that the small numbers of Vγ5^+^ DETCs present in *Tnfrsf11a*^−/−^ mice epidermis were also Vδ1^+^, suggesting that residual levels of *Skint1* detected in the absence of Rank may still support the generation of small numbers of invariant DETCs. Nevertheless, the combined data on *Tnfrsf11a*^−/−^ mice, demonstrating a defect in intrathymic Vγ5^+^ thymocyte maturation, coupled to diminished numbers of Vγ5^+^ DETCs, demonstrate that efficient maturation of an invariant Vγ5TCR^+^ DETC repertoire depends upon expression of the TNFSF-Receptor Rank, just as it depends on Skint-1 (Barbee et al., 2011; Lewis et al., 2006).

### Skint-1-Mediated Vγ5^+^ DETC Development Proceeds Normally in *Aire*-Deficient Mice

**Figure 7:**
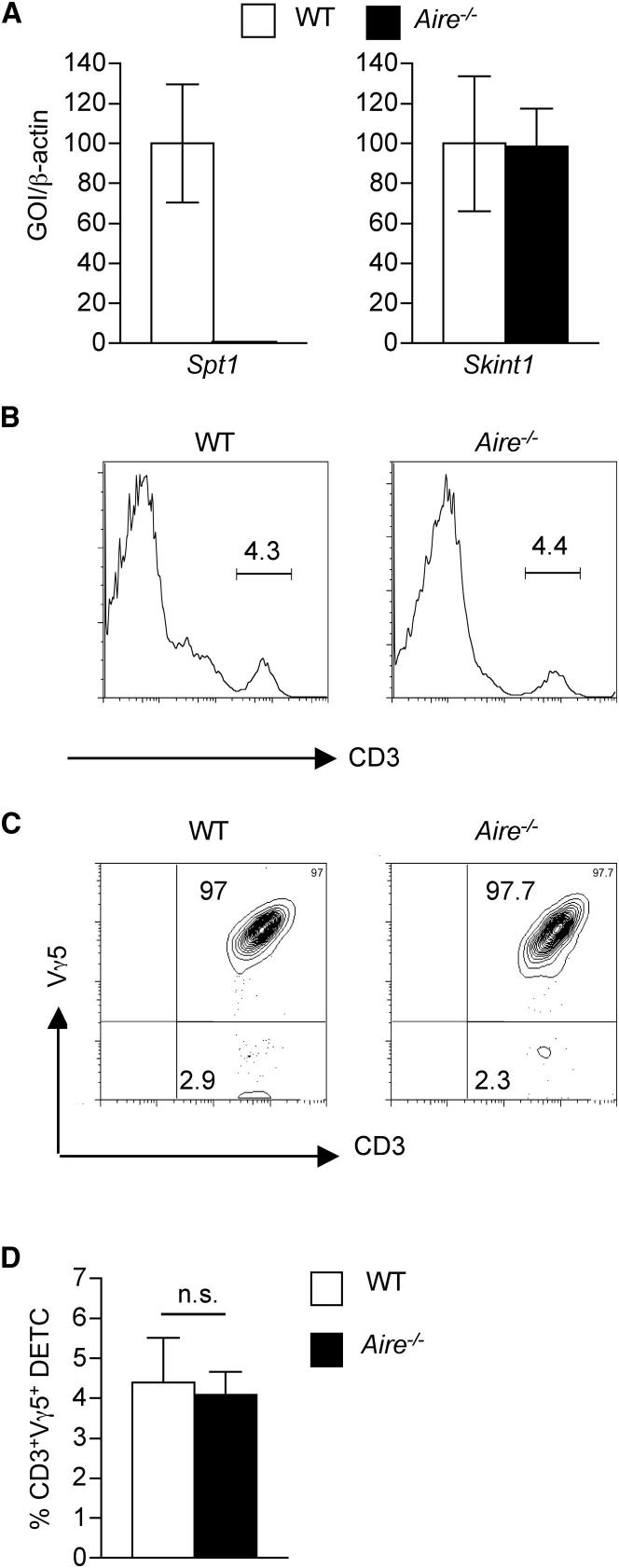
Skint-1 Expression Occurs Independently of Aire, and *Aire*^−/−^ Mice Display Normal Development of Invariant Vγ5^+^ DETCs (A) Quantitative PCR analysis of expression of the indicated genes in neonatal thymuses from WT (white bars) and *Aire*^−/−^ (black bars) mice. (B and C) Epidermal preparations from WT and *Aire*^−/−^ neonates were analyzed by flow cyometry for the presence of CD3^+^ T cells (B), together with antibodies to the Vγ5TCR (C). (D) Frequency of CD3^+^Vγ5^+^ DETCs in WT (white bars) and *Aire^−/−^* (black bars) neonates. n.s. is not significant.

To investigate whether the importance of Rank-dependent Aire^+^ mTEC development for Skint-1-mediated DETC maturation is directly dependent upon Aire itself, we analyzed *Skint1* expression and the emergence of epidermal Vγ5^+^ DETCs in neonatal *Aire*-deficient mice. We found that in contrast to the known Aire dependency of genes such as *Spt1* (salivary protein 1) (Figure 7A), *Skint1* expression was unaltered in *Aire*^−/−^ neonatal thymus (Figure 7A). In line with this lack of requirement for Aire in the expression of *Skint1*, comparison of the neonatal epidermis of *Aire*^−/−^ and WT mice showed no changes in the frequency of epidermal CD3^+^ cells (Figure 7B), including those expressing Vγ5^+^ (Figures 7C and 7D). Thus, despite the association between the maturation of Aire^+^ mTECs and invariant Vγ5TCR^+^ DETC maturation, *Aire* deficiency does not impair the emergence of an invariant Skint-1-dependent DETC pool.

## Discussion

Intrathymic medullary microenvironments are known to play an important role in establishing tolerance in newly generated αβ T cells (Anderson et al., 2007; Takahama, 2006). In particular, medullary epithelial cells that express the *Aire* gene have been shown to participate in T cell tolerance induction in several ways (Mathis and Benoist, 2009). For example Aire^+^ mTECs have been directly linked to the negative selection of single-positive thymocytes that bear αβTCRs recognizing tissue-restricted antigens (Liston et al., 2003), as well as the development of FoxP3^+^ natural regulatory T cells (Aschenbrenner et al., 2007), and most recently in the intrathymic positioning of XCR1^+^ dendritic cells (Lei et al., 2011). Thus, the establishment of medullary thymic areas containing Aire-expressing epithelial cells represents an important and multifaceted component of intrathymic tolerance mechanisms. Of equal importance, recent data show that the nascent cohorts of Aire^+^ mTECs that are generated in the fetal and neonatal periods are both essential and sufficient for tolerance induction (Guerau-de-Arellano et al., 2009). Moreover, although shaping of the αβ T cell repertoire fosters Aire^+^ mTEC differentiation in the adult thymus (Hikosaka et al., 2008; Irla et al., 2008), the initial emergence of Aire^+^ mTECs during these key developmental stages occurs independently of, and prior to, the generation of mature thymocytes by positive selection (Derbinski et al., 2001; White et al., 2008; Zuklys et al., 2000). Collectively, these observations suggest the existence of an alternative, perhaps developmental stage-specific, mechanism that enables functionally competent intrathymic medullary microenvironments to be in place prior to αβ T cell selection events, where they impose tolerance induction on the first cohorts of newly generated T cells.

Here, we show that initial thymus medulla formation, involving generation of the first cohorts of Aire^+^ mTECs, occurs as a result of a cellular combination of innate LTi cells and progenitors of an invariant T cell (DETC) subset that is defined by invariant expression of the Vγ5Vδ1TCR. Indeed, we found that *Tcrd^−/−^* mice lack a significant fraction of the Aire^+^ mTEC compartment and that DETC progenitors express Rankl, a known regulator of Aire^+^ mTEC development. Our finding that both immature CD45RB^lo^ and mature CD45RB^hi^ Vγ5^+^ thymocytes express Rankl suggests that the ability of Vγ5^+^ thymocytes to influence mTEC development is not limited to the mature stages of their development. This is in contrast to the impact made by αβ T cells, which require continued maturation from the CD4^+^CD8^+^ to the CD4^+^ or CD8^+^ stage to express Rankl (Hikosaka et al., 2008). Rather, a scenario can be envisaged that during hematopoietic crosstalk for the initiation of mTEC development, and within individual medullary areas of embryonic thymus, simultaneous input from LTi and Vγ5^+^ thymocytes can occur, rather than a temporal sequence that first involves LTi cells and then mature Vγ5^+^ thymocytes, the latter generated as a result of Skint-1-mediated differentiation. Interestingly, although analysis of *Tcrd*^−/−^ × *Rorc*^−/−^ double-deficient mice showed a further reduction in the frequency of Aire^+^ mTECs as compared to single mutants, small numbers of Aire^+^ mTECs remain. Whether these cells arise a result of the early emergence of mature Rankl^+^αβTCR^+^ thymocytes that are already known to influence mTEC development or through Rankl provision by an additional hematopoietic cell type is currently unknown. Whatever the case, our finding suggests that, at this early developmental stage, LTi and γδ T cells combine to influence thymus medulla formation.

That initial embryonic thymus medulla formation is influenced by invariant DETC progenitors appears relevant to their highly selective and timed appearance in thymus ontogeny at around E14–E16 (Havran and Allison, 1988), parallel to the emergence of the first Aire-expressing medullary environments. It is currently unclear whether the ability of cells of the γδ T cell lineage to direct Aire^+^ mTEC development is limited to those expressing the Vγ5TCR. However, it is interesting to note that γδTCR^+^ thymocytes in the adult also express Rankl (Hikosaka et al., 2008), and mTEC numbers are reduced in adult *Tcrd^−/−^* mice (Hikosaka et al., 2008). Thus, in the adult thymus, αβTCR^+^ and γδTCR^+^ thymocytes may synergize to regulate thymus medulla development.

Importantly, the present results also show that the development of Vγ5^+^ DETC progenitors and Aire^+^ mTECs in the fetal thymus are reciprocally linked, in that the intrathymic crosstalk of the two cell types is required to generate epithelial environments that support and select γδ T cells. Thus, we show that Rank-mediated stimulation of immature mTECs induces expression of *Skint1*, and that in the absence of Rank expression there is a block in the maturation of Vγ5TCR^+^ thymocytes and a corresponding increase in the frequency of CD3^+^ epidermal-resident DETC-bearing TCRs encoded by other Vγ gene segments. Collectively, these findings therefore establish a new role for intrathymic expression of Rank in the development of invariant γδ T cells, related to its capacity to promote maturation of mTECs, the intrathymic source of Skint-1. However, despite their similar expression patterns, *Skint1* gene expression in mTECs is Aire independent and the invariant DETC T cell pool is intact in neonatal *Aire*^−/−^ mice. Although γδ T cells are not grossly affected in the context of *Aire* deficiency in mouse and man (Tuovinen et al., 2009), the possibility that there are alterations in the fine specificities of diverse, adaptive γδ T cells merits further study. Thus, the induction of Aire^+^ mTECs by the first wave of γδ T cells may limit the emergence of potentially harmful, self-reactive γδTCR^+^ and αβTCR^+^ cells.

## Experimental Procedures

### Mice

The following mice were used in this study: C57BL/6, *Tcrd*^−/−^ (Itohara et al., 1993), *Rorc*^−/−^ (Sun et al., 2000), *Tnfrsf11a*^−/−^ (Rossi et al., 2007), *Relb*^−/−^ (Weih et al., 1995), and *Aire*^−/−^ (Ramsey et al., 2002). *Tcrd*^−/−^ and *Rorc*^−/−^ were intercrossed at The University of Birmingham to generate *Tcrd*^−/−^ × *Rorc*^−/−^ double knockout (DKO) mice. All mice were bred and maintained at the Biomedical Services Unit, University of Birmingham, except for *Aire*^−/−^ mice, which were housed at Biomedical Services, John Radcliffe Hospital, University of Oxford. For the generation of timed pregnancies, day of detection of a vaginal plug was designated day zero. All experiments were performed under the authorization of the UK Home Office.

### Antibodies and Immunoconjugates

The following antibodies were used for flow cytometry: anti-TCRVγ5 (clone 536, BD PharMingen), anti-Vγ5Vδ1 (clone 17D1), anti-CD3 (clone 145.2C11, BD PharMingen), anti-CD205 (clone NLDC-145, Abcam), anti-CD40 (clone 3/23, BD PharMingen), anti-EpCAM1 (clone G8.8, kind gift of A. Farr, University of Washington), anti-CD45 (clone 30-F11, eBioscience), anti-CD45RB (clone C363.16A, eBioscience), anti-Aire (clone H512, kind gift of H. Scott, Adelaide University), anti-Ly51 (clone 6C3, eBioscience), and anti-CD80 (clone 16-10A1, eBioscience). Biotinylated antibodies were revealed with streptavidin conjugated to Alexa Fluor 555. The following additional primary antibodies were used for confocal analysis: anti-medullary epithelium (clone ERTR5, kind gift of W. van Ewijk), anti-CD8 (clone CT-CD8b, eBioscience), anti-RORγ (clone AFKJS-9, eBioscience), anti-IL-7Rα (clone A7R34, eBioscience), pan-γδTCR (clone GL3, eBioscience), and goat anti-Aire (D-17, Santa Cruz Biotechnology). For detection of polyclonal goat anti-Aire, anti-goat Alexa Fluor 594 (Invitrogen) was used. For detection of ERTR5 antibody, anti-Rat IgM Alexa Fluor 594 (Invitrogen) was used. Detection of RORγ Abs was achieved with anti-rat IgG FITC (Jackson Immunoresearch), then anti FITC-Alexa Fluor 488 (Invitrogen), and finally anti-rabbit IgG-Alexa Fluor 488 (Invitrogen).

### Real-Time PCR Analysis

cDNA was obtained from purified mRNA with μMacs One-step cDNA synthesis kit, according to the manufacturer's instructions (Miltenyi Biotec). Real-time PCR was performed with SYBR Green with primers specific for *ACTB* (β-actin), *Aire*, *Spt1* (salivary protein 1), and *Skint1* on the Rotor-Gene-3000 PCR machine (Corbett Research, NSW, Australia). PCRs were conducted in replicates in 15 μl volumes in reaction buffer containing 1× SensiMix QPCR SYBR No ROX Mix (Quantace) and 200 nM of primers for *Aire*, *Rankl*, *Spt1*, and *Skint1*; *ACTB* primers were designed and synthesized as a Quantitect Primer Assay (QIAGEN). After an initial denaturation step (95°C for 10 min), cycling was performed at 95°C for 15 s, 60°C (*ACTB*, *Spt1*, and *Skint1*) or 62°C (*Aire* and *Rankl*) for 20 s, and 72°C for 15 s (40 cycles). Specific amplification was verified by melt curve analysis. Reaction amplification efficiency and the Ct values were obtained from Rotor Gene 6.0 software (Corbett Research) with standard curves generated from Mouse Universal cDNA Reference-oligo dT primed (Biochain Institute). Calculation of the relative mRNA expression values for each sample normalized to *ACTB* was performed as described with the Pfaffl quantitation method (Pfaffl, 2001). Fold levels represent the mean (±SEM) of replicate reactions and data shown are representative of at least two independent experiments. Information on primers is summarized in Table S1.

### Flow Cytometry and Cell Sorting of Thymic Stromal Cells and Thymocytes

Multicolor flow cytometry was performed as described (Shakib et al., 2009) with BD-LSR and BD-Fortessa machines (BD Biosciences); cell sorting was performed with a Beckman Coulter XDP MoFlo (Beckman Coulter). To perform intracellular staining of Aire, RTOCs were disaggregated with 0.25% trypsin then surface stained with anti-CD45, anti-Ly51, and anti-eEpCAM1. Cells were washed in PBS, incubated in IC Fixation buffer (eBioscience) at 4°C for 60 min, and then washed twice in permeabilization buffer (eBioscience). A staining solution of Alexa Fluor 488-conjugated anti-Aire in permeabilization buffer was added to cells, after incubation for 30 min at 4°C. Cells were washed in permeabilization buffer twice then analyzed by flow cytometry. CD3^+^Vγ5^+^ thymocytes were purified from E15 thymus lobes explanted in organ culture for 7 days. In some experiments, total CD3^+^Vγ5^+^ thymocytes were sorted into immature and mature subsets on the basis of low and high levels of CD45RB, respectively. LTi were prepared from organ-cultured E15 fetal spleens as described (Rossi et al., 2007), and both CD4^+^CD8^+^ thymocytes and CD4^+^CD8^−^αβTCR^hi^ thymocytes were sorted from mechanically disrupted adult thymus preparations. To isolate TEC subsets for qPCR analysis, CD40^−^CD205^+^ immature cTECs (Shakib et al., 2009) were MoFlo sorted from total EpCAM1^+^CD45^−^ cells from E16 thymus, whereas total EpCAM1^+^CD45^−^ cells from E15+7d FTOCs were used to isolate CD40^+^CD205^+^ mature cTECs, CD80^+^CD205^−^ mature mTECs, and CD80^−^CD205^−^ immature mTECs (Shakib et al., 2009).

### Confocal Microscopy and Quantitation

Images were obtained with a LSM 780 microscope (Zeiss) and analyzed with Zen software (Zeiss). For images with six different stains, expression of ERTR5 from a serial section was imported with Zen software. For quantitation, medullary areas were measured with Zen software and cells of a given phenotype counted.

### Fetal Thymus and Reaggregate Thymus Organ Culture

Freshly dissected E15 fetal thymus lobes were placed in organ culture conditions for between 5 and 7 days, as described (Shakib et al., 2009). 1.35 mM 2 deoxyguanosine was added to cultures to deplete hematopoeitic cells (Jenkinson et al., 1992); in some experiments, anti-Rank (10 μg/ml, R&D Systems) was added for a further 3 days to induce Aire^+^ mTEC development (Rossi et al., 2007). To prepare reaggregate thymus cultures (Jenkinson et al., 1992), 2-dGuo-treated thymus lobes were trypsinized, depleted of remaining CD45^+^ cells, and then mixed at a 5:1 ratio with either freshly prepared Vγ5^+^ thymocytes or LTi cells. The resultant cell suspension was then deposited onto the surface of a 0.8 μm Nucleopore filter in organ culture. At the indicated time point, RTOCs were disaggregated with 0.25% trypsin/0.02% EDTA and analyzed by flow cytometry. For experiments involving blockade of Rankl, RTOCs were established with either Vγ5 thymocytes or LTi, as described above, and recombinant OPG (R&D Systems) was added to cultures at a final concentration of 10 μg/ml.

### Analysis of DETCs in Epidermal Sheets

For FACS analysis, skin from the backs of neonatal (between 0 and 2 days) mice was placed dermal side down in 20 mM EDTA (Sigma) at 37°C for 2 hr. Epidermal sheets were then peeled from the dermis, washed in PBS, and incubated in 1 mg/ml Collagenase D (Roche) and 40 μg/ml DNase (Sigma) for 1 hr. Cells were filtered and stained for anti-TCRVγ5 FITC (clone 536, BD Biosciences) and anti-CD3 APC (clone145.2C11 eBioscience).
