# Supplementary material for: Rank Signaling Links the Development of Invariant γδ T Cell Progenitors and Aire+ Medullary Epithelium
Source: Immunity. 2012 Mar 23;36(3-2):427–37. doi: 10.1016/j.immuni.2012.01.016 (PMC3368267; doi:10.1016/j.immuni.2012.01.016)
Supplement: Document S1. Table S1 [file mmc1.pdf]

Immunity, Volume *36*

Supplemental Information

Rank Signaling Links the Development of Invariant γδ T Cell Progenitors and Aire^{+} Medullary Epithelium

Natalie A. Roberts, Andrea J. White, William E. Jenkinson, Gleb Turchinovich, Kyoko Nakamura, David R. Withers, Fiona M. McConnell, Guillaume E. Desanti, Cecile Benezech, Sonia M. Parnell, Adam F. Cunningham, Magdalena Paolino, Josef Penninger, Katja Simon, Takeshi Nitta, Izumi Ohigashi, Yousuke Takahama, Jorge H. Caamano, Adrian C. Hayday, Peter J.L. Lane, Eric J. Jenkinson, and Graham Anderson

Table S1. Primer Sequences for qPCR Analysis

Primer sequences, NCBI Reference Sequence Accession Numbers and amplicon sizes are as follows:

Gene	NCBI Reference Sequence	Forward Sequence (5'---3')	Reverse Sequence (5'---3')	Amplicon size (bp)
Aire	NM_009646.1	TGCATAGCATCC TGGACGGCTTCC	CCTGGGCTGGAG ACGCTCTTTGAG	187
Rankl	NM_011613.3	CACACCTCACCA TCAATGCTGC	GAAGGGTTGGAC ACCTGAATGC	394
Salivary protein-1	NM_009267.2	GGCTCTGAAACT CAGGCAGA	TGCAAACTCATC CACGTTGT	304
Skint-1	NM_001102662.1	TTCAGATGGTCA CAGCAAGC	GAACCAGCGAAT CTCCATGT	143
β-actin	NM_007393	QuantiTect Mm_Actb_1_SG PRIMER Assay (Qiagen QT00095242)		149
